# Nasal Reconstruction in Granulomatosis with Polyangiitis Using an L-shaped Cartilage Strut Graft

**DOI:** 10.1007/s00266-025-04892-y

**Published:** 2025-05-20

**Authors:** Lorand Imre Czimbalmos, Zsofia Bere, Laszlo Kovacs, Peter Kovacs, Laszlo Rovo, Gabor Vass

**Affiliations:** 1 Department of Oto-Rhino-Laryngology and Head-Neck Surgery, University of Szeged, Tisza Lajos krt. 111, Szeged, 6725 Hungary; 2 Otorhinolaryngology and Head and Neck Surgery Clinic, University of Debrecen, Nagyerdei krt. 98, Debrecen, 4032 Hungary; 3 Department of Rheumatology and Immunology, University of Szeged, Kálvária sgt. 57, Szeged, H-6725 Hungary; 4 Department of Dermatology and Allergology, Plastic Surgery Ward, University of Szeged, Korányi fasor 6, Szeged, 6720 Hungary

**Keywords:** Granulomatosis with polyangiitis, WG-Wegener’s granulomatosis, Rhinoplasty, Cartilage strut graft, Nasal reconstruction

## Abstract

**Methodology:**

Patients exhibited progressive saddle nose deformity as a result of GPA. To reconstruct the nose, a single-stage open rhinoplasty was conducted involving the placement of an independent L-shaped costal cartilage implant. Graft formation and fixation were performed according to the author-developed method.

**Results:**

From 2012 to 2023, seven patients with severe saddle nose deformity underwent implantation of modified L-shaped costal cartilage strut grafts. Except for one complicated case, all patients reported satisfaction with the surgical outcomes.

**Conclusions:**

In this study, the visual appeal of the nose and the respiratory capabilities of the patients improved significantly. The L-shaped costal cartilage strut graft is an effective technique for saddle nose deformity correction. This technique provides excellent outcomes, including improved nasal function and aesthetics. Further studies with a larger sample size are needed to confirm these findings.

**Level of Evidence II:**

This journal requires that authors assign a level of evidence to each article. For a full description of these Evidence-Based Medicine ratings, please refer to the Table of Contents or the online Instructions to Authors  www.springer.com/00266.

## Introduction

Saddle nose deformity is characterized by the structural collapse or depression of the nasal bridge, leading to a flattened or a saddle-shaped appearance. This deformity has multiple causes, such as trauma, infections, autoimmune diseases, congenital problems or nose surgery [1]. Infections such as syphilis or leprosy can also destroy the nasal cartilage [2], and congenital abnormalities such as congenital chondrodysplasia punctata can disrupt the development and growth of the nasal cartilage and bone, resulting in the collapse of the nasal dorsum [3, 4].

Such a condition can be a result of the excessive removal of or a change in the nasal cartilage during rhinoplasty or recurrent surgical interventions, such as revision rhinoplasties, which can compromise the support nasal structures.

Granulomatosis with polyangiitis (GPA), previously known as Wegener’s granulomatosis, is a rare, severe and frequently life-threatening systemic autoimmune disease characterized by the presence of antibodies targeting several cytoplasmic components in neutrophil granulocytes. It is also characterized by necrotizing inflammation of capillaries, arterioles and venules and predominantly affects upper airway, kidney and lung functions [5]. The average age of the patients at diagnosis ranged from 40 to 60 years, with no significant difference between men and women. The annual incidence rate is approximately 0.5–15 cases per million individuals. In 90% of the cases, the nasal mucosa is affected, resulting in a characteristic phenotype known as “Wegener’s nose.” Relapsing polychondritis, another immune-mediated rheumatic disease with nasal involvement, causes inflammation results in cartilage destruction without affecting the mucosa, eventually causing the collapse of nasal structures. Nevertheless, the distorted appearance of the nose is a mental burden for patients, and the structural deterioration of the cartilaginous framework and the nasal cavity adversely affects breathing [6]. In these cases, nasal reconstruction is difficult because of the near-complete absorption of the nasal septum and the absence of significant support from the septal cartilage in the nasal dorsum and columella. In addition, the mucoperichondrium is destroyed by inflammation; therefore, mucosal pocket formation for the graft is challenging. In addition, no extensive data support the reconstruction of saddle nose deformities in patients with GPA, even though a recent review on the topic included findings from a maximum of 36 individuals. In addition, autologous rib cartilage is a commonly used graft because a robust piece can be harvested in one block. Histologically, a stable hyaline cartilage can be modeled well [7].

At our department, a two-piece, L-shaped costal cartilage strut (L-CCS) graft is ultimately applied for the reconstruction of saddle nose deformities in patients with GPA. In the past 10 years, we consistently followed a standardized approach for our reconstructive procedure, ensuring that the surgical stages and graft shaping were uniform. This extensive follow-up study aimed to reveal the outcomes of our standardized approach. Thus, to assess both the functional and aesthetic results, feedback from patients was collected using a previously introduced quality of life survey.

## Materials and Methods

### Patients

Between 2012 and 2022, seven Caucasian patients with GPA (2 males and 5 females) presented with advanced saddle nose deformities in our clinic. Based on Daniel’s saddle nose classification (1), four patients had type III deformities and three had type IV deformities (Table 1).

**Table 1:** Study patient population

Patient no.	1	2	3	4	5	6	7
Sex.	Male	Male	Female	Female	Female	Female	Female
Saddle nose deformity	IV	III	III	IV	IV	III	III
Age at surgery	17	25	24	29	23	21	53
Operation time (minutes)	90	180	60	60	90	120	120
Immunosuppressive therapy administered	Rtx, Mtx	Rtx, Cycl	Rtx, Mtx, Cycl	Rtx, Mtx, Cycl	Plasmapheresis, Cycl, Azathioprin	Cycl, Azathioprin	Rtx
Corticosteroids administered (i.v.)	Methylprednisolone	Methylprednisolone	Methylprednisolone	Methylprednisolone	Methylprednisolone	Methylprednisolone	Methylprednisolone
No. of relapses	–	1	–	–	–	–	–
Complications	–	–	–	–	–	–	–
Preoperative ROE	1	1	1	2	1	1	1
Postoperative ROE	5	5	3	5	5	4	5

*Cycl*. Cyclophosphamide, *Mtx* methotrexate, *ROE* rhinoplasty outcome evaluation, *Rtx* rituximab

Saddle nose deformity grading based on Daniel’s saddle nose classification, where type 0 (pseudosaddle), type I (minor-cosmetic concealment), type II (moderate-cartilage vault restoration), type III (major-composite reconstruction), type IV (severe-structural reconstruction), and type V (catastrophic-nasal reconstruction)

Previously, GPA was histopathologically confirmed in all patients. To determine the optimal timing for surgical treatment, the selection criteria also encompassed strict rheumatological assessments. Consequently, all patients attended preoperative consultations with rheumatologists and ear, nose and throat (ENT) specialists. The remission of the GPA was verified using laboratory tests and local examination. Patients exhibiting an active disease or displaying systemic signs or active organ involvement were not eligible for reconstruction. Signs of mucosal inflammation, massive purulent discharge or acute signs of upper airway infection were also an exclusion factor. However, patients on maintenance immunosuppressive treatment without symptoms were not disqualified. Each patient provided informed consent, demonstrating their willingness to undergo the surgery.

### Surgery and Postoperative Follow-up

**Fig. 1:**
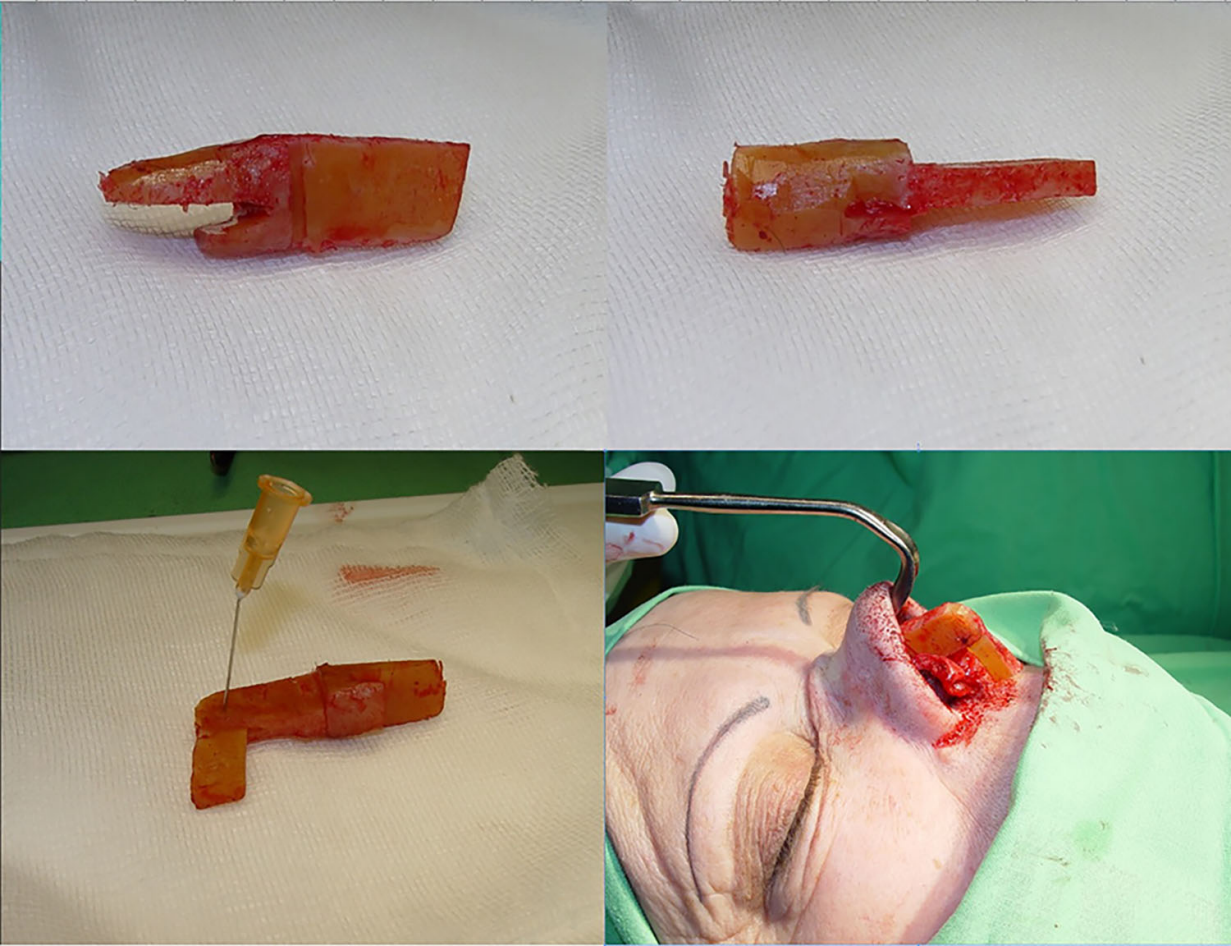
L-shaped costal cartilage graft harvested and shaped to the needs of the final position, sutured with PDS, fitted to the created graft pocket

**Fig. 2:**
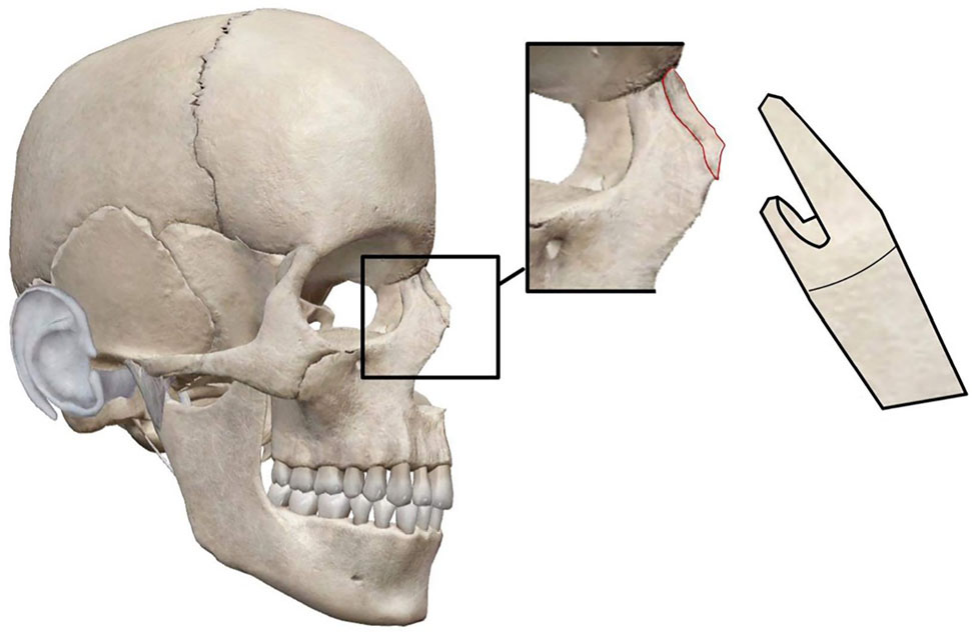
Graft placement. The cranial end of the dorsal section is sculpted into a rectangular notch, which precisely fits the lamina of the bony pyramid eliminating the need for additional fixation sutures

For reconstruction, one-stage open rhinoplasty with autonomous L-shaped costal cartilage implantation was performed. The costal cartilage was taken from a small (5–10 cm) submammary incision made at the level of the 6 th–7 th rib cartilage. The total length of the nasal dorsum and columella was measured, and the required length of the graft was approximated. The distal section of the rib cartilage, i.e., the cartilage section close to the cartilage–rib bone joint, was removed with the perichondrium (Fig. 1). Using prior measurement as a reference, an autologous cartilage graft was made and inserted into the pocket between the skin and the nasal mucosa of the columella and dorsum (Fig. 2). Before the development of L-CCS grafts, alternative methods, such as onlay graft and spreader graft techniques, are used to reconstruct saddle nose deformities using rib cartilage [20]. However, these techniques had limitations, including graft instability and insufficient projection of the nasal tip.

**Fig. 3:**
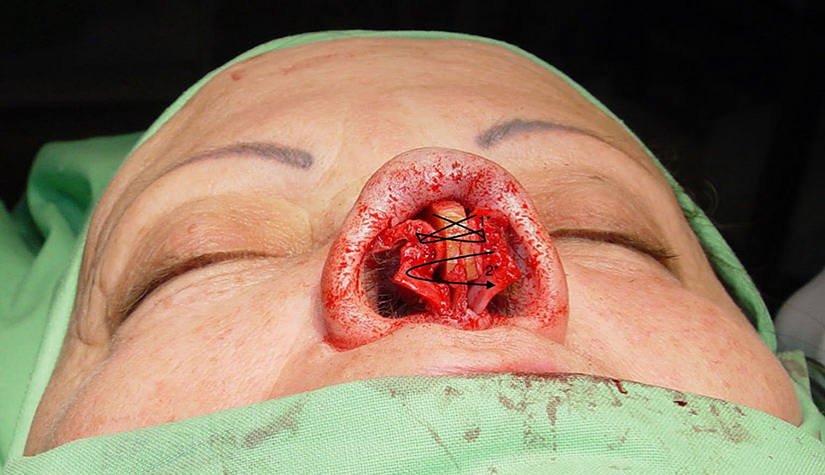
Graft attachment to the alar cartilages (as shown in this figure) with transdomal (1*) and interdomal (2*) sutures, securing both the lateral and medial parts of the alar process

The L-CCS graft was designed to overcome these limitations. The method involves partitioning the rib cartilage into two segments, with one being of longer than the other. The longer segment offers structural reinforcement for the nasal dorsum, whereas the shorter portion provides both support and projection for the nasal tip. The columellar strut part is positioned on the anterior nasal spine utilizing a small groove created in the lower section of the cartilage and then secured with PDS sutures (as seen in the bottom right image in Fig. 1). The graft is attached to the alar cartilages (as shown in Fig. 3) with transdomal (1*) and interdomal (2*) sutures, thereby securing both the lateral and medial parts of the alar process, ensuring sufficient space for the nasal inlet and giving it a symmetrical shape. The cranial end of the dorsal section is sculpted into a rectangular notch, which precisely fits the lamina of the bony pyramid, eliminating the need for additional fixation. The two-cartilage grafts are attached in the tip region using the “tongue in the groove technique” and secured with PDS sutures (Fig. 1).

For the keystone area, the dissection is performed in the sub-SMAS (superficial muscular aponeurotic system) layer of the soft tissue while maintaining the integrity of the remaining septal mucoperichondrium. The periosteum of the remaining nasal bone does not require elevation at the cranial end of the graft due to the rectangular notch technique previously described. In cases where fixation of the caudal end of the graft to the anterior nasal spine presents challenges, a drilled hole in the spine suffices for precise positioning. Thereafter, meticulous contouring of the framework is executed using a nasal (Fomon) rasp to eliminate the level difference until the transition becomes unnoticeable to ascertain the ultimate form of the nose. The incisions were then closed. For thoracic wounds, a 12-G drain was kept in place until the amount of fluid draining from the wound consistently remained >10 mL. During the early postoperative stage, considering that all patients were immunosuppressed, intravenous antibiotics (amoxicillin and clavulanic acid) were administered to avoid superinfection and nonsteroidal anti-inflammatory drugs (diclofenac) to manage pain. Oral antibiotic therapy continued for another 7 days after discharge. All patients were examined by an ENT expert on days 7 and 14 post-emission, when the stitches and nasal casts were removed. The frequency of follow-up has been biannual, except for the 2020–2021 period because of SARS-CoV-2 restrictions.

### Data Collection

Preoperatively, the following demographic and medical data of each patient were collected: age, sex, age at GPA onset, remission onset and currently used medications.

Regarding surgical data, the surgical length, intraoperative complications, duration of hospitalization, early and late postoperative complaints and wound healing tendency were recorded. During follow-up, flare-ups of GPA signs, if there were, with special attention to the involvement of the nasal cavity, and applied immunosuppressive medication were also recorded.

The adapted rhinoplasty outcome evaluation (ROE) questionnaire presented previously by our group [8] was applied: The same four questions were asked before and after surgery, and the patient had to score each question on a scale of 0–4, where 0 and 4 points represent the least and the highest satisfaction, respectively:

(1) How much do you like the appearance of your nose? (2) How much can you breathe through your nose? (3) How much do you think your friends and those close to you like your nose? (4) Do you think the appearance of your nose limits your social or professional activities?

The average pre- and postoperative scores for each question were compared using the nonparametric paired sample test, i.e., Wilcoxon test, in IBM SPSS Statistics for Windows version 20 (IBM Corp., Armonk, NY, USA), and a p value of 0.05 was considered significant.

### Statistical Analysis

Data are presented as mean ± standard deviation unless otherwise stated. Mean values were compared using the Wilcoxon rank-sum test.

## Results

In our patient population, the age at GPA onset varied from 12 to 56 years (range, 17–55 years) at the time of surgery. For immunosuppressive therapy, rituximab, methotrexate, or cyclophosphamide was administered either previously for induction treatment or as maintenance therapy at the time of surgery, which was used in combination in most cases, and plasmapheresis was also performed in one case (Table 1).

The average surgical time was 120 ± 10 min. No intraoperative complications, such as severe bleeding or pneumothorax, occurred. The median hospitalization time was 5 (5 ± 2) days.

Until emission, no severe pain (visual analog scale [VAS] score of 4.5 ± 2, where 0 and 10 represent no pain and worst pain possible, respectively) or wound healing problems were observed. On days 1 and 2 (i.e., days 7 and 14 post-emission), pain reduced to a VAS score of 2.5 ± 1.

The pre- and postoperative results of the ROE questionnaire are presented in Table 2. The average postoperative outcomes, assessed 3 months post-surgery, showed significant improvement over the preoperative scores (*p* = 0.00156, Wilcoxon rank-sum test) (Table 2).

**Table 2:** Data collected from the rhinoplasty outcomes evaluation (ROE)

	Preoperative		Postoperative	
	Mean ± SD	Median	Mean ± SD	Median
How much do you like the appearance of your nose?	0 ± 0	0	4.9 ± 0.4	5
How much can you breathe through your nose?	3 ± 0.8	3	3.6 ± 0.8	3
How much do you think your friends and those close to you like your nose?	0.7 ± 0.8	1	5 ± 0	5
Do you think the appearance of your nose limits your social or professional activities?	1 ± 1.2	1	4.7 ± 0.5	5

Unfortunately, a 27-year-old female patient exhibited symptoms of soft tissue infections on the recipient site, leading to the dehiscence of the incision line, purulent discharge and cartilage graft denudation. A bacterial sample was collected from the purulent discharge, and intravenous antibiotic therapy was initiated, amoxicillin–clavulanic acid, which was continued based on the resistance profile of *Staphylococcus aureus*. In addition, topical antibiotics were administered. Rheumatology consultations and laboratory tests were performed to rule out the presence of acute GPA. Despite extensive therapeutic intervention and consistent wound management, cartilage grafts at the columella and a significant amount of the nasal dorsum were absorbed, leading to the recurrence of saddle nose deformity.

**Fig. 4:**
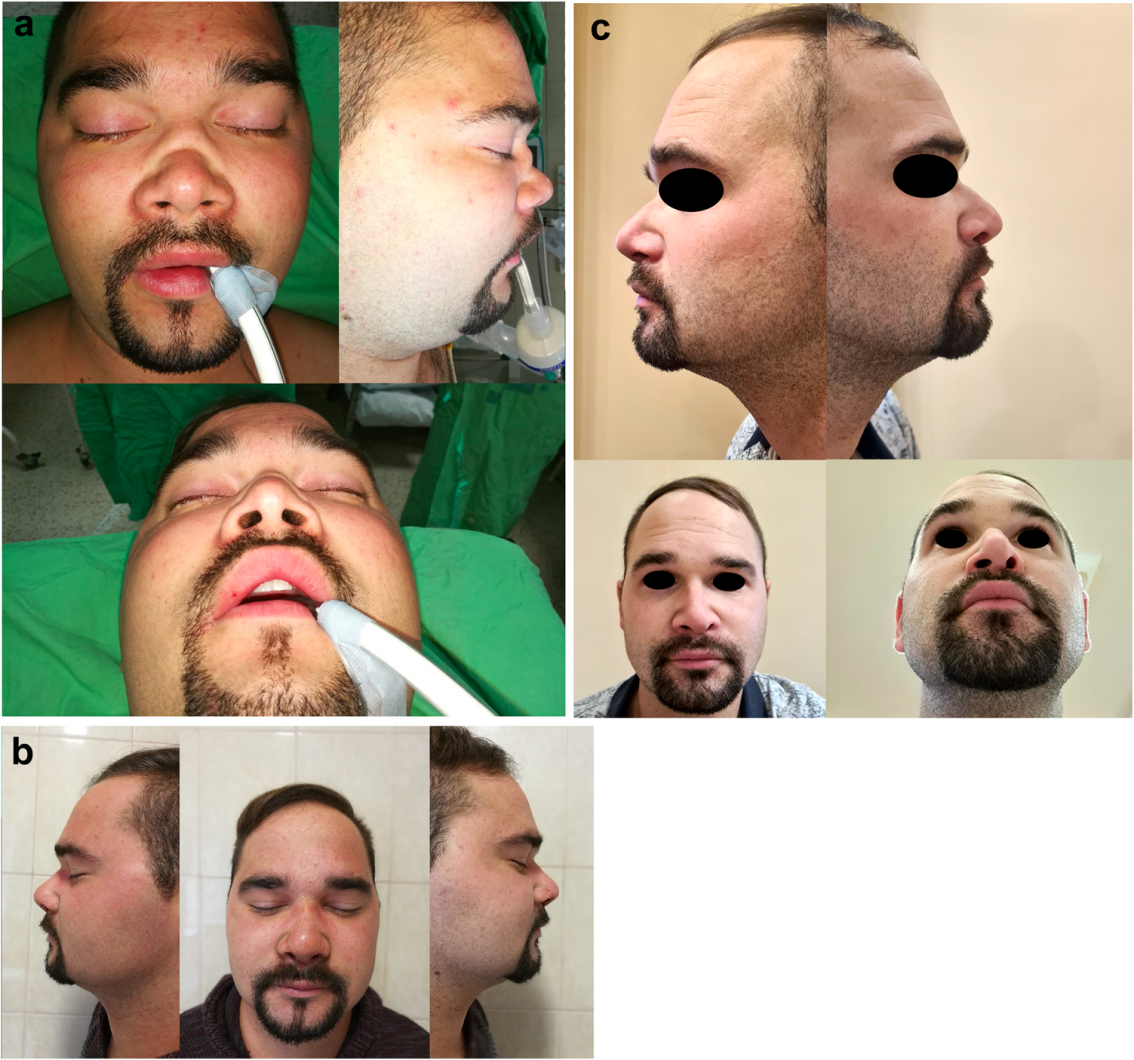
**a** Appearance of a male patient preoperatively and **b** 6 months postoperatively, **c** 5 years postoperatively

In one case (an 18-year-old male), 3 years postoperatively, acute flare-up of GPA caused a relapse with paranasal and tympanic progression along with subglottic granuloma formation. Consequently, further immunosuppressive treatment (rituximab) was required. Despite the acute phase, no nasal involvement was detected, and the graft survived completely, without any major aesthetic or functional deterioration; even after 5 years, the graft remains in place and is functioning perfectly (Fig. 4a,b,c).

**Fig. 5:**
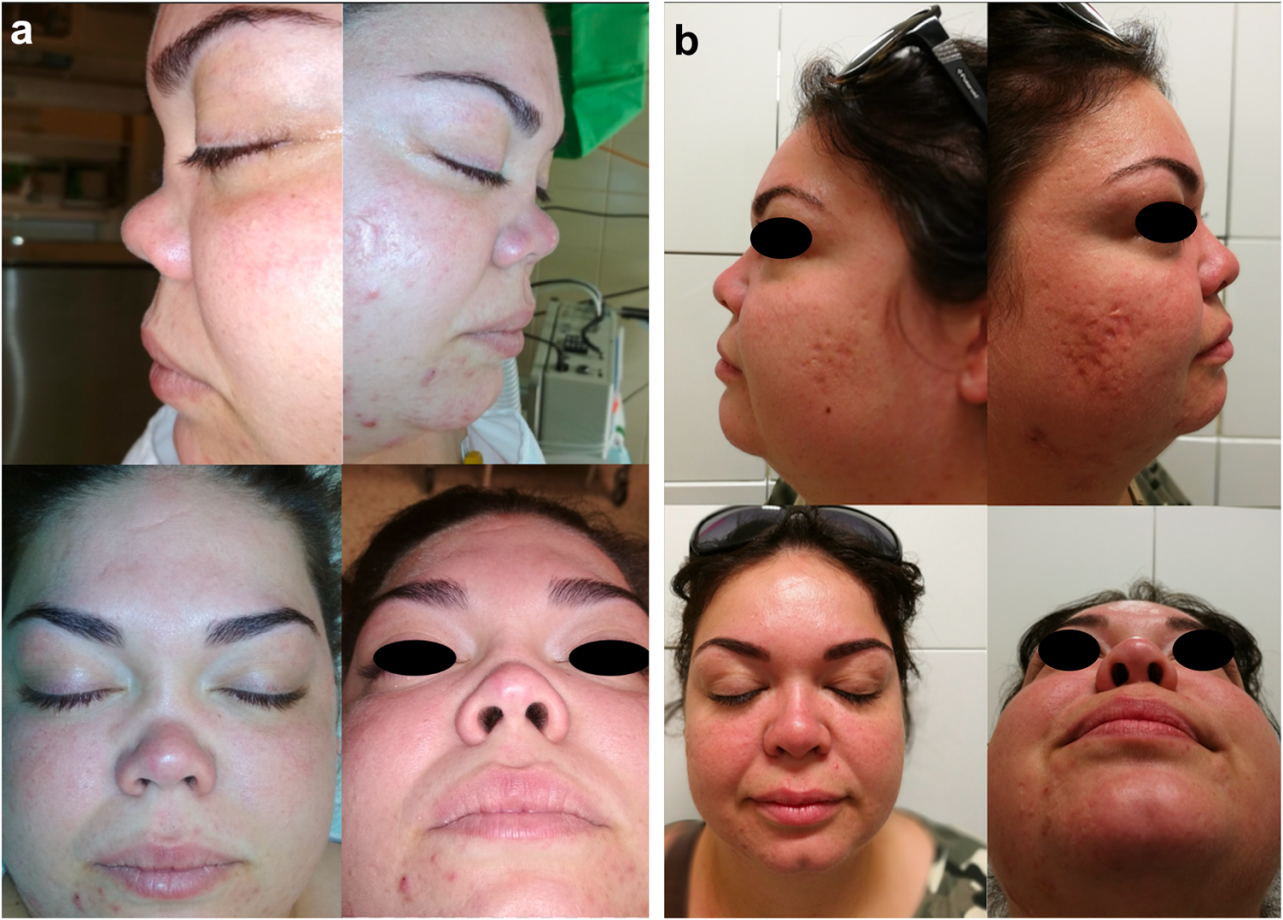
**a** Appearance of a female patient preoperatively and **b** 6 months postoperatively

Except for the single case of postoperative local infection described earlier, the modified ROE questionnaire administered at the second assessment revealed noteworthy improvements in all aspects. Specifically, the postoperative scores for both functional and aesthetic factors surpassed the preoperative scores (Table 2). The extended monitoring period revealed a sustainable and pleasing aesthetic outcome in these cases. The follow-up period ranged from 1 to 10 years, with an average duration of 60 months. During this period, personal assessments were conducted every 6 months, except for the 2020–2021 period, which was affected by SARS-CoV-2-related restrictions. The conclusive follow-up occurred in 2023 (Fig. 5a,b).

## Discussion

GPA, historically referred to as Wegener’s granulomatosis, is a potentially fatal disease mostly affecting the upper and lower respiratory system and the kidneys, resulting in the development of necrotizing granulomatous lesions [9]. The ENT is frequently affected in patients with GPA, ranking as the most frequently involved organs upon diagnosis [10]. In many instances, the lesions in these areas manifest before any respiratory or renal symptoms can be recognized. The prevalence and intensity of ENT symptoms highlight the need to assess the effect of GPA on patients, given that 70–95% of people with GPA experience head and neck symptoms. Furthermore, the inflammation frequently causes irreversible damage to the upper airway tissues [11]. The nasal cavity and paranasal sinuses are the most frequently affected locations, with a prevalence of 64–80%. This is followed by symptoms in the ear, pharynx and larynx [12]. Typical sinonasal symptoms consist of nasal crusting, chronic rhinosinusitis symptoms, nasal obstruction and nasal necrotic–bloody discharge [13]. Typically, the anterior part of the septum is affected, including the vascular plexus that provides blood to the septal components. This leads to serious damage to the cartilaginous and bony structures [14]. Biopsy usually reveals necrotizing granulomas with giant cells and vasculitis that is predominantly characterized by neutrophils. However, all diagnostic symptoms uncommonly occur simultaneously [9]. The clinical presentation includes the presence of mucosal erosions, scar development, occurrence of septal perforation and distinct deformity characterized by a reduction in the height of the nasal dorsum and a reduced nasal length [12]. The symptomatic category of additional ENT sites includes various conditions, such as tinnitus, otitis media, otitis externa, tympanic membrane perforation, mastoiditis, sensorineural or conductive hearing loss and subglottic stenosis as an airway manifestation [12]. Without systemic treatment, the condition can be fatal because it involves the lower airway, kidneys and other organs [15]. However, in most cases, permanent remission can be achieved with immunosuppressive therapy [16], and saddle nose deformity remains a permanent stigma for patients. Although surgical nasal reconstruction exists, the procedure has not yet become widespread. Initially, patients are mostly monitored by immunologists and rheumatologists, who may not be familiar with this rehabilitation. Furthermore, the procedure requires surgical staff experienced in the reconstructive technique and can adjust to any potential intraoperative circumstances. Moreover, GPA recurrence and the potential subsequent deterioration of the rebuilt nose are prevalent concerns that hinder surgeons from initiating the surgery for such malformations.

In this study, we presented a standardized method to reconstruct the saddle nose deformity in GPA. To ensure the optimal outcome of augmentation rhinoplasty, the complex nature of these deformities requires careful consideration of perioperative factors, and a multidisciplinary perioperative approach is necessary to create a safe and individualized service for patients [17]. Patients with autoimmune disorders require special consideration from a rheumatologist to oversee disease progression and preoperative evaluation by an anesthesiologist and otolaryngologist to rule out airway abnormalities, such as subglottic and tracheobronchial stenosis.

In this patient cohort, it is crucial to consider that significant improvement in nasal breathing cannot be achieved due to the nature of the disease. The nasal cavity mucosa is destroyed, and the turbinates, which play a role in airflow perception, are absent. Patients experience a phenomenon similar to paradoxical nasal obstruction following radical mucotomy, where despite a widened nasal cavity due to partial turbinate removal, obstruction symptoms persist due to the absence of turbinate mucosa and its associated mechanoreceptors. This complex interplay between anatomical changes and sensory perception highlights the challenges in treating such conditions. The loss of turbinate tissue not only affects the physical structure of the nasal passages but also disrupts the intricate sensory mechanisms that contribute to the subjective feeling of nasal patency. As a result, patients may continue to experience sensations of nasal obstruction even when objectively, their nasal passages appear more open. Furthermore, the surgical intervention does not influence the crusting process, which is a hallmark of the underlying disease. This persistent crusting can further compromise nasal airflow and contribute to ongoing breathing difficulties. Consequently, the quality of nasal breathing depends not only on the surgery but also on regular nasal toileting. This emphasizes the importance of post-operative care and patient compliance in managing symptoms [18].

John Orlando Roe published the first article on the treatment of saddle nose deformity in 1887 and titled “The deformity termed'pug-nose'and its correction by a simple operation.” In 1892, Robert F. Weir initially tried to cure saddle nose deformity by transplanting the sternum of a duck into the contracted nose of a patient with syphilis. In 1896, Israel pioneered the utilization of human bone grafts in the nasal region [1]. Over the past 20 years, several graft materials, including homologous calvarial bone, autologous iliac crest and ear cartilage, have been examined. Currently, the use of autologous costal cartilage graft is the most commonly used procedure for repair [19]. The utilization of the rib cartilage for nasal reconstruction has been practiced since the early twentieth century. However, the specific technique of using an L-shaped costal cartilage strut (L-CCS) graft was first documented in 1999 by David B. Lovice, Dean M. Toriumi and his colleagues [20].

This method yields exceptional results, encompassing enhanced nasal functionality and attractiveness. Since its inception, the L-CCS graft is becoming increasingly popular as an effective approach for treating saddle nose deformity. Surgeons have made adjustments to the original approach to enhance the results [22–24]. These variations involve altering the graft size and shape and using additional grafts for support and fixation. The uniqueness of our approach lies in our attempt to minimize the required manipulation and stitching of the graft while ensuring a secure positioning simultaneously. Our long-term follow-up analysis has also demonstrated that even with robust graft size relative to the anatomy, it consistently provides outstanding aesthetic results and maintains adequate breathing function.

Considering that infection is the primary risk for graft rejection and absorption [25], in addition to parenteral antibiotic treatment, preparing graft pockets is also essential. Openings in the mucosa must be avoided because this can lead to bacterial contamination of the grafts within the nasal cavity.

Stability, i.e., resistance of such grafts, is also a major concern in GPA [26]. Permanent low immunosuppressive therapy and long-term corticosteroid administration can affect wound healing [14] and immune reactions against postoperative bacterial superinfection, particularly when the operation is performed in a nonsterile environment, such as the airway or nasal cavity. According to the data, surgery should be performed with a modest steroid dosage because steroids tend to hinder wound healing [27–29]. Nonetheless, extended nonsteroidal immunosuppressive drug therapy is necessary for disease management and cannot be excluded. Considering our patient demographic, we assert that perioperative systemic and supplementary local antibiotic treatments are crucial to prevent such problems. In addition, surgery must be avoided during the initial phase of severe inflammation or symptomatic GPA because it may exacerbate wound healing issues due to higher doses of immunosuppressive or steroid medications. Furthermore, it poses an unnecessary risk in terms of anesthesia. However, the stability of the graft remains intact even with a relapsing GPA. To our knowledge, our study obtained a greater number of cases than research utilizing similar methodologies [14]. Our results align with those of studies that have examined similar patient populations. Unlike previous sporadic evidence, our strategy may also minimize complications [25, 26, 30].

Rib cartilage grafts have emerged as a cornerstone in rhinoplasty, particularly for intricate cases and revisions, yet their propensity for warping remains a notable challenge. To combat this issue, several esteemed rhinoplasty surgeons have pioneered innovative techniques. Jack Gunter introduced the use of K-wires for internal stabilization, while Bahman Guyuron championed meticulous carving and shaping of costal cartilage grafts. Rod Rohrich has been at the forefront of concentric carving research and has extensively explored rib cartilage applications in rhinoplasty. The arsenal of warping-reduction techniques has expanded to include the oblique split method, balanced cross sections, delayed implantation, and opposing suture techniques. These approaches have shown promising results in maintaining graft stability over time. In cases of saddle nose deformity, where substantial augmentation is often necessary, rib cartilage grafts have proven particularly advantageous due to their abundant volume and structural integrity [31].

While these considerations are crucial for patients with GPA, the field of rhinoplasty offers various graft options for nasal reconstruction, each with its own set of advantages and limitations. In the field of rhinoplasty, hyaluronic acid (HA) offers immediate, reversible results with high patient satisfaction, though its effects are temporary and carry potential vascular risks. Fat grafting provides natural augmentation with dual benefits of volume enhancement and skin rejuvenation, but faces challenges of unpredictable resorption and potential complications such as fat necrosis. Cadaveric costal cartilage (CC) emerges as a viable alternative to autologous grafts, avoiding donor-site morbidity while demonstrating low warping and infection rates comparable to autologous cartilage. However, CC usage is limited by potential resorption risks, especially in irradiated grafts, and higher costs associated with procurement and storage. Each option presents unique advantages and limitations, with HA being suitable for minor corrections and temporary results, fat grafting for natural-feeling augmentation with potential regenerative benefits, and CC for structural support in major reconstructions. The choice among these options should be tailored to individual patient needs, considering factors such as desired longevity of results, extent of required correction and willingness to undergo repeated procedures or more invasive surgeries [32, 33].

Saddle nose deformity, particularly in complex cases with immunological illness, can be effectively treated using an L-CCS graft. The proposed method offers a straightforward and effective approach to creating an optimal L-cartilage graft for nasal reconstruction and enhancement of its stability, resulting in admirable aesthetic and functional outcomes without compromising the risk of oculonasal synkinesis or significant voice alterations postoperatively [34–36]. Nevertheless, a multidisciplinary approach and the cooperation of immunologists and rheumatologists are vital to GPA management.

The limitations of this study primarily involve the difficulty of presenting a large, homogeneous patient cohort due to the relatively small number of cases. This challenge is compounded by the fact that both immunologists managing the underlying condition and patients themselves may not fully recognize the potential for reconstruction or the likelihood of favorable outcomes. Immunologists, naturally, prioritize the treatment of the primary disease, with reconstruction only considered viable following successful management of the underlying pathology. Nonetheless, the number of cases we report is noteworthy compared to existing literature. It is also crucial to underscore the importance of performing such reconstructive procedures in specialized centers to facilitate the development of an appropriate learning curve, which is essential for achieving consistent and high-quality outcomes.

Looking ahead, our research aims to broaden the patient cohort while maintaining regular follow-up with existing patients, ultimately intending to publish these findings.
